# Production and characterization of absorbent heat from the bark of residual Brazil nut bark (*Bertholletia Excelsa l.*)

**DOI:** 10.1186/s13065-015-0114-3

**Published:** 2015-06-16

**Authors:** Selma dos Santos Melo, Joel Estevão de Melo Diniz, Jonilson Heslei Guimarães, Josivan da Silva Costa, Davi do Socorro Barros Brasil, Sílvia Simone dos Santos de Morais, Daímio Chaves Brito, José Carlos Tavares Carvalho, Cleydson Breno Rodrigues dos Santos, Denilson Luz da Silva

**Affiliations:** Laboratory of Environmental Processes, Federal University of Pará, Rua Augusto Corrêa, 01, Belém, Pará 66075-110 Brazil; Laboratory of Physical Chemistry, State University of Amapá, Av. Presidente Vargas, Campus I, 650, Macapá, Amapá 68900-070 Brazil; Laboratory of General and Analytical Chemistry, Federal University of Amapá, Rod. JK Km 2, s/n, Macapá, Amapá 68902-280 Brazil; Institute of Technology, Federal University of Pará, Rua Augusto Corrêa, 01, Belém, Pará 66075-110 Brazil

**Keywords:** Absorbent, Brazil nut, Thermal activation

## Abstract

**Background:**

The increasing efforts to reduce the environmental impact on the Amazon’s natural resources are focusing on watercourses that pass through effluents with high concentrations of heavy metals. The adsorption by absorbent is one of the methods used to remove metallic ions. In this assignment, the preparation of activated carbon from Brazil nut bark *(Bertholletia excelsa l.)*, which is a waste material produced from the use of seeds in foodstuffs and cosmetics, is shown.

**Results:**

The absorbent was carbonized at 400 °C in 3 h and activated at 800 °C in 2 h, having received the name of AC2, and, the specific area, pore size, real and apparent densities, porosity, scanning electron microscopy (SEM) coupled to energy-dispersive X-ray spectroscopy (EDS), X-ray diffraction (XRD), pH, moisture, fixed carbon and surface functional groups by Boehm method and Fourier transformed infrared spectroscopy (FTIR) were characterized. According to the results, the carbon presented alkaline characteristic, mesoporosity, average pore diameters of 2.203 nm and specific surface area by BET of 464.835 m^2^ g^-1^.

The efficiency of removal was performed in synthetic solutions of copper sulphate (II) pentahydrate (CuSO_4._5H_2_O), evaluating the influence of pH, initial concentration of copper solution (II), particle diameter and time contact of the adsorbent in solution. The results of higher removal percentages were to pH 5.09, initial concentration of 50, 100 and 150 mg^-1^ diameter 0.595 < D < 1.19 mm and time contact of 5 min.

**Conclusions:**

The Brazil nut bark is shown to be an important bio-waste, being an excellent alternative material for the low-cost production of activated carbon for use in processes involving iterations of adsorption.

## Background

One of the main problems causing environmental pollution of watercourses is industrial effluents that have high concentrations of heavy metals, with disagreements existing on the maximum values allowed by current legislation. The search for practical, efficient and low cost alternatives has been a constant to circumvent these problems.

Some of the many processes employed to reduce the concentrations of heavy metals in industrial effluents are adsorption, ion exchange, membrane separation and electrochemistry, and these often become economically unviable because they are costly to industries, and are thus not a priority to invest in as they are processes that may affect profit and production.

Among various techniques, the alternative that has attracted considerable attention is adsorption, since it presents itself as an efficient and economical method in wastewater treatment [1, 2].

Adsorption is an effective technique for the purification and dehydration of gases and as a means of fluid fractionating, which are difficult to separate by other means of separation [3]. This process occurs when a molecule of solute, present in a solution, accumulates on the surface of a solid due to the action of unbalanced surface forces [4, 5]. The molecules which are adsorbed at the solid/liquid interface are termed adsorbate, while the solid material is adsorbent [6].

Absorbents are natural or synthetic substances with crystalline structure presenting a high surface area for a given mass. The inner surface of the pore is accessible to a selective adsorbent-adsorbate combination [7].

Among the most commercially used adsorbents one finds activated charcoal, zeolites, silica gel and activated alumina, owing to their high surface areas.

Activated charcoal or activated carbon (AC) is a general name given to a group of carbonaceous materials manufactured to have a highly developed porosity and high surface area. Due to its large adsorption capacity, it is a material that has long been used as an adsorbent to purify, detoxify, filter, discolor, separate or concentrate liquid and gaseous materials in diverse areas such as the food, pharmaceutical, chemical, oil, nuclear and automotive industries, and is also applied in wastewater treatment industries and in the process of potability of water [8].

The first step of activated carbon preparation is the carbonization, which is usually performed in the absence of air at temperatures of 400 to 800 °C. During the carbonization of raw materials, condensation of polynuclear aromatic compounds and the breaking of groups of the chemical chain occurs, resulting in a carbon residue. In this environment, cross-linked reactions take place, which inhibit the development of pre-graphite structure [9]. Two methods can be employed for the activation of charcoal: activation by a chemical process or activation by thermal process, also called physical activation.

In chemical activation, the carbonization and activation occur in a single step, in which the vegetal precursor impregnated with a suitable chemical agent (H_2_SO_4_, H_3_PO_4_, ZnCl_2_, alkali metals hydroxides etc.), is charred. The great advantage of chemical activation is related to the low energy cost, temperatures in the range 400–800 °C, and high process efficiency. Various studies of chemical activation by inorganic agents such as phosphoric acid, potassium hydroxide and ZnCl_2_, which have been used in the activation of precursors of agricultural waste, are found in the literature [10].

In thermal activation, AC is produced from material that has been carbonized, that is, a precursor already heat treated. The carbonized material is activated at temperatures that may range 600–1200 °C in a flux of water steam or carbon dioxide, or a mixture of both, for between 1 and 10 h. The gasification removes the carbonaceous material within the particles, resulting in the creation of pores and clearing of existing ones, leading to a development of the porous structure of the material [11].

Numerous studies have been performed using residues of Amazon natural plants in the production of activated carbon. Agricultural residues are the most common materials studied for this purpose, since they are renewable, usually available in large quantities and cheaper than other materials used to manufacture a variety of adsorbents [12–14]. According Capobianco et al. (2004) [15], it is possible to develop activated carbon from biomass with pores of nanometer dimensions. The use of biomaterial reduces environmental impact in two ways: removal of the residual biomass, which is a pollutant, from where it was generated or deposited and treatment of the contaminated effluent with this residue [16]. Thus, it becomes feasible to use alternatives to biomass residues, such as the Brazil nut shell, coconut shell, acai berry lump, buriti lump, and cupuaçu peel among others [17].

The Brazil-nut or Pará-nut is the seed of the chestnut tree (*Bertholletia excelsa L*.) of the botanical family *Lecythidaceae*, gender *Bertholletia*, and in consideration of the majesty of its bearing it was called *excelsa* (species). Exploiting Brazil-nuts became the main economic activity of the Amazon region, following the decline in the exploitation of rubber. This activity has sustained thousands of extractive and a whole oligarchy due to its value [18].

This paper aims to use the residual biomass of Brazil-nut for the preparation of physically activated carbon (absorbent) at a temperature of 800 °C within 2 h. The prepared charcoal had its chemical and physical structure characterized. The proposal to transform these shells into activated carbon may create an ecologically viable destination for these wastes.

## Experimental

### Preparation of adsorbent

The raw material used in preparing the samples of absorbent were the shells of the Brazil-nut (*Bertholletia excelsa*), a residual material of chestnut processing. Firstly, this material was selected, washed in running water and then dried in an oven (drying and sterilization), model 315 SE of the brand FANEM-Brazil, at a temperature of 105 °C ± 5 for 24 h. After drying, the material was placed on two ceramic vases, where were charred and calcined in the muffle-type electric furnace of QUIMIS model Q318M24-Brazil.

### Production and characterization of AC2

#### Carbonization

Carbonization assays of AC2 were performed in 10 runs and each in duplicate, wherein the previously dried shells were stored in ceramic vessels of 10.8 cm and 11.8 cm in diameter and carbonized at 400 °C ± 1 for 3 h in a muffle furnace, with a mean heating rate of 17 °C min^-1^. The masses obtained at the beginning and end of the procedure were important for determining yield parameters on charcoal (ACY) and on coal volatile material (CVM) released during the carbonization according to the methodology of Ramos (2005) [10].

#### Thermal activation

In this step, the same ceramic vessels were used, this time with materials charred in the previous step and inserted in the muffle furnace for activation during 2 h at 800 °C ± 1, in average time of 57 min, with a mean heating rate 17 °C min^-1^. The mass obtained at the beginning and at end of the procedure were recorded to determine the parameters of average yield of activated charcoal (AYAC) and volatile material of activated charcoal (VMAC) released during calcination in accordance with Ramos (2005) [10].

#### Characterization techniques

AC2 was characterized as: moisture by ASTM D 2867-04 method, ash content by ASTM D 2866-94 method, pH by ASTM 3838-05 method, porosity in fixed bed ABNT NBR 9165-1985, bulk density according to ASTM D 2854-09 method, actual density and fixed carbon performed according to Ramos (2005) [10].

#### Specific surface area

The analysis of the specific surface area was obtained by the theory of multilayer in which nitrogen was used in its gaseous phase N_2_ at 77 K, with approximately 40 adsorption-desorption cycles. The AC2 samples were treated at the temperature of 250 °C for 2 h before each test. The obtained data were analyzed by BET (Brunauer, Emmet and Teller) method, using a porosimeter of type MICROMERITICS TRISTAR-II USA.

#### Porosimetry

Data of the mean diameter and total pore volume were obtained from the N_2_ adsorption isotherm in gas phase at 77 K with adsorption-desorption cycles by the method of Barrett, Joyner and Halenda (BJH) using a MICROMERITICS TRISTAR-II USA porosimeter.

#### Scanning electron microscopy (SEM) coupled with Energy dispersive X-ray spectroscopy (EDS)

The SEM analysis was performed on electron microscope, model LEO-1430-USA; conditions for images of secondary electrons (ES) with beam current of 90 μA at constant voltage of 20 kV, working distance of 15 mm; AC2 samples were coated with a thin layer of platinum in sputter Emitech K550-USA.

#### X-ray diffraction

The analysis for phase identification was carried out on the samples by the total powder method using an X-ray diffractometer model X’PERT PRO MPD of PANALYTICAL- USA, with a goniometer PW 3050/60 (θ-θ) with X-ray ceramic tube with copper anode Cu (Kα_1_ = 1.540598 Å), model PW 3373/00 with fine focusing, Ni Kβ filter, 2200 W, 40 kV and 40 mA. For samples,the following were used: a sweep angle from 5 ° to 75 °, a voltage of 40 kV and current of 30 mA, a step size of 0.0170 ° and a time/step of 10 s, and a fixed divergent and anti-scatter.

#### Surface functional groups

The surface chemical properties of charcoal were determined by the acidity or basicity, which can be altered when in liquid and gaseous phases if oxidising agents exist in its structure, which when treated with solutions such as nitric acid, sodium hypochlorite or hydrogen peroxide modify the nature and the amount of oxygen in the complex surface of charcoal [9]. The surface functional groups of AC2 were determined by Fourier transform infrared spectroscopy, (FTIR) and by Boehm method [19–22]. FTIR was carried out using the Thermo Scientific Nicolet apparatus, model IS10-USA in the region 4000-400 cm^-1^. The carboxylic groups were obtained in tests with potassium hydrogen carbonate (KHCO_3_). The amount of phenolic groups were found by the difference between groups found in titration tests with sodium hydroxide (NaOH) and sodium carbonate (Na_2_CO_3_), and lactonic groups by the difference of the groups found in tests with sodium carbonate (Na_2_CO_3_) and potassium hydrogen carbonate (KHCO_3_). To calculate the mass of acidic surface functional groups by Boehm method, Equation 1 was used [19–22]:

1 $$ ASFG=\frac{0,1\mathrm{x}\kern0.5em f\kern0.5em \mathrm{x}\left({T}_b-T\right)\mathrm{x}\left(50/20\right)}{w} $$

Where: T_b_, the volume of HCl 0.1 mol L^-1^ consumed by the solution of NaOH 0.1 molL^-1^ for the blank experiment (mL); T, the volume of HCl 0.1 mol L^-1^ consumed in different filtered solutions after the time of contact with AC (mL); f, the factorization of HCl 0.1 mol L^-1^ and w , the mass of AC used (g).

#### Influence of pH on the adsorption of copper (II)

To determine the influence of pH in solution in the adsorption process, the copper (II) solution was previously adjusted with standard solutions of HCl and NaOH 0.1 mol.L^-1^, and the measured pHs into a potentiometer of countertop HANNA in the ranges of: 3.4; 4.01; 5.09 and 6.01. The experiments were performed in duplicate for each pH range, keeping constants the initial concentration (50 mg L^-1^ of Cu II), volume of the solution (100 mL), solution temperature (27.2 °C), particle diameter (0.595 < D < 1.19 mm), mass in grams of the adsorbent (1.0 g) and agitation frequency (150 rpm). Then the samples were filtered and stored for determining the final concentration by atomic absorption.

#### Influence of CA2 particle diameter in adsorption of copper (II)

To determine the effect of CA2 particle diameter, granulometric studies were performed using sieves with mesh 14, 28 and 48 (1.19 mm, 0.595 mm and 0.297 mm) according to the Brazilian Association of Technical Norms (ABNT, Associação Brasileira de Normas Técnicas). Assays were performed in duplicate where the particle size varied within a range of 0.595 to 1.19 mm (D > 1.19 mm; 0.595 < D < 1.19 mm and D < 0.595 mm). For this study, initial concentration (50 mg.L^-1^ of Cu II), volume of the solution (100 mL), solution pH (5.47), solution temperature (27.2 °C), mass in grams of the adsorbent (1.0 g), contact time (60 min) and agitation frequency (150 rpm) remained constant. The samples were then filtered and stored for determining the final concentration by atomic absorption.

#### Influence of the contact time of CA2 in solution in the adsorption of copper (II)

For studying the contact times of CA2 assays were performed in duplicate and adsorption times evaluated at 1, 2, 5, 8, 10, 20, 30, 60, 90 and 120 min. The initial concentration (50 mg.L^-1^ of Cu II), volume of the solution (100 mL), solution pH (5.47), Solution temperature (27.2 °C), particle diameter (0.595 < D < 1.19 mm), mass in grams of the adsorbent (1.0 g) and agitation frequency (150 rpm) were maintained as constants. The samples were then filtered and stored for determining the final concentration by atomic absorption.

#### Influence of the equilibrium concentration

To study the equilibrium concentration in the adsorption process using CA2 as the adsorbent to remove Cu (II) of aqueous solution, the adsorption assays were performed in duplicate. For these experiments, 1.0 g of charcoal with diameter 0.595 < D < 1.19 mm were used, which were placed in Erlenmeyer of 250 mL containing 100 mL of aqueous solution of Cu (II) at the initial concentrations 5, 10, 20, 30, 50, 100, 150 and 200 mg.L^-1^ with pH equal to 5.46, temperature 27.2 °C and contact time of 60 min. The flasks were closed with a plastic film and excited at a frequency of 150 rpm. After said time, the samples were filtered for the determination of final concentration by atomic absorption. With equilibrium concentration data from all the tests it was possible to calculate the percentage of removal of Cu (II) by Equation (2):

2 $$ \mathrm{R}\left(\%\right)=\left[\frac{{\mathrm{C}}_{\mathrm{i}}\ \hbox{-}\ {\mathrm{C}}_{\mathrm{e}}}{{\mathrm{C}}_{\mathrm{i}}}\right]\mathrm{x}100 $$

Where C_i_: initial concentration of Cu (II) (mg.L^-1^) and C_e_: final concentration or equilibrium concentration Cu (II) (mg.L^-1^).

With regard to the study of the amount of copper (II) adsorbed by mass of charcoal at equilibrium, the equation (3) was used:

3 $$ \mathrm{Q}\mathrm{e}=\frac{\left(\mathrm{Ci}\ \hbox{-}\ \mathrm{C}\mathrm{e}\right)\ \mathrm{x}\ \mathrm{V}}{\mathrm{M}} $$

Where Q_e_: amount of Cu (II) adsorbed (mg adsorbate/g adsorbent);

C_i_: initial concentration of Cu (II) (mg L^-1^);

C_e_: final concentration or equilibrium concentration of Cu (II) (mg L^-1^);

V: volume of the solution (L) and M: mass of charcoal CA2 (g).

## Results and discussion

The quality of absorbent is determined by its physical and chemical properties: density, moisture and chemical composition (fixed carbon, ash and volatiles). Table 1 shows the results of the experimental tests of characterization of the AC2. As might be expected, charcoal presented basic characteristics after having been subjected to an activation temperature of 800 °C. The basic absorbent developed basic oxides on its surface and in its ashes, which are responsible for the rise in pH when in solution, and thus adsorb acidic compounds [23].

**Table 1:** Characterization of activated charcoal from Brazil-nut bark

Properties	Results	Standart Deviation
Volatile material of charcoal (%)	49.2823	3.6226
Yield of charcoal (%)	50.7176	3.6226
Volatile material of activated carbon(%)	71.8145	3.6389
Yield of activated charcoal (%)	28.1855	3.6389
Moisture (%)	8.0385	0.2154
Ash (%)	4.8475	0.0858
Fixed carbon (%)	23.3379	0.5813
pH	10.10	0.0141
Real density (g cm^-3^)	0.9556	0.0028
Bulk Density (g cm^-3^)	0.4728	0.0089
Porosity (%)	50.5209	0.9254

Results of AC2 properties were very close to the values of charcoals from other vegetable precursors presented in Table 2. The absorbents were characterized by a large surface area in the range 300–4000 m^2^ g^-1^ evaluated by the BET method and are higher when mixed with solvent.^7^ Specific surface areas of the AC2 were found in the range of particle sizes of 200 mesh, and analyzed by BET (S_BET_) method, relative pressure (S_P/P°_) and Langmuir (S_Langmuir_). The results obtained were 464.835 m^2^ g^-1^ specific surface (BET method).

**Table 2:** Characterization of area, volume e diameter of AC2

Parameters	Mean Values f	Deviation
BET Specific Area - S_BET_ (m^2^.g^-1^)	464.835	9.7735
Specific Area of a single point in P/P° - S_P/P°_ (m^2^ .g^-1^)	477.6824	0.2044
Langmuir Specific Area- S_Langmuir_ (m^2^ .g^-1^)	619.9551	3.8205
Pore volume - V_P/P°_ (cm^3^ g^-1^)	0.2561	(-)
Accumulated volume of pores in the adsorption - V_BJH-Ads_ (cm^3^ g^-1^)	0.0662	(-)
Accumulated volume of pores in desorption - V_BJHDes_ (cm^3^ g^-1^)	0.0731	(-)
Mean pore diameter -D (nm)	2.2031	(-)
Mean pore diameter in adsorption - D_BJH-Ads_ (nm)	4.9227	(-)
Mean pore diameter in desorption - D_BJH-Des_ (nm)	4.0695	(-)

**Fig. 1:**
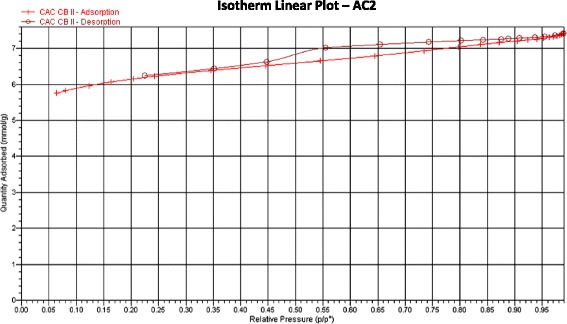
Adsorption/desorption isotherm of N_2_ by AC2 at 77 K

AC2 is evenly comprises mostly mesopores with an average diameter of 2.2031 nm shown in Table 2. Mean diameter and total pore volume were determined by the BJH method (Barret, Joyner and Halenda), through gas adsorption analysis using N_2_ at 77 K to obtain a full isotherm (adsorption/desorption) of N_2_ with partial pressures ranging from 0.06 P/P° to 0.99 P/P° (Fig. 1), which when compared with the Brunauer classification is classified as a type II isotherm [24].

Type II isotherm is characteristic of a physical or reversible type adsorption, and tend to form multilayer adsorption. It is observed in the isotherm a loop similar to the hysteresis type H4, which is related to the mechanism of condensation of N_2_ in the mesopore. According to the IUPAC classification, this H4 hysteresis loop is associated with the presence of wedge-shaped micro and meso pores, cones and/or parallel plates and cylindrical capillaries open at both ends [25].

### Scanning electron microscopy (SEM) coupled with Energy dispersive X-ray spectroscopy (EDS)

**Fig. 2:**
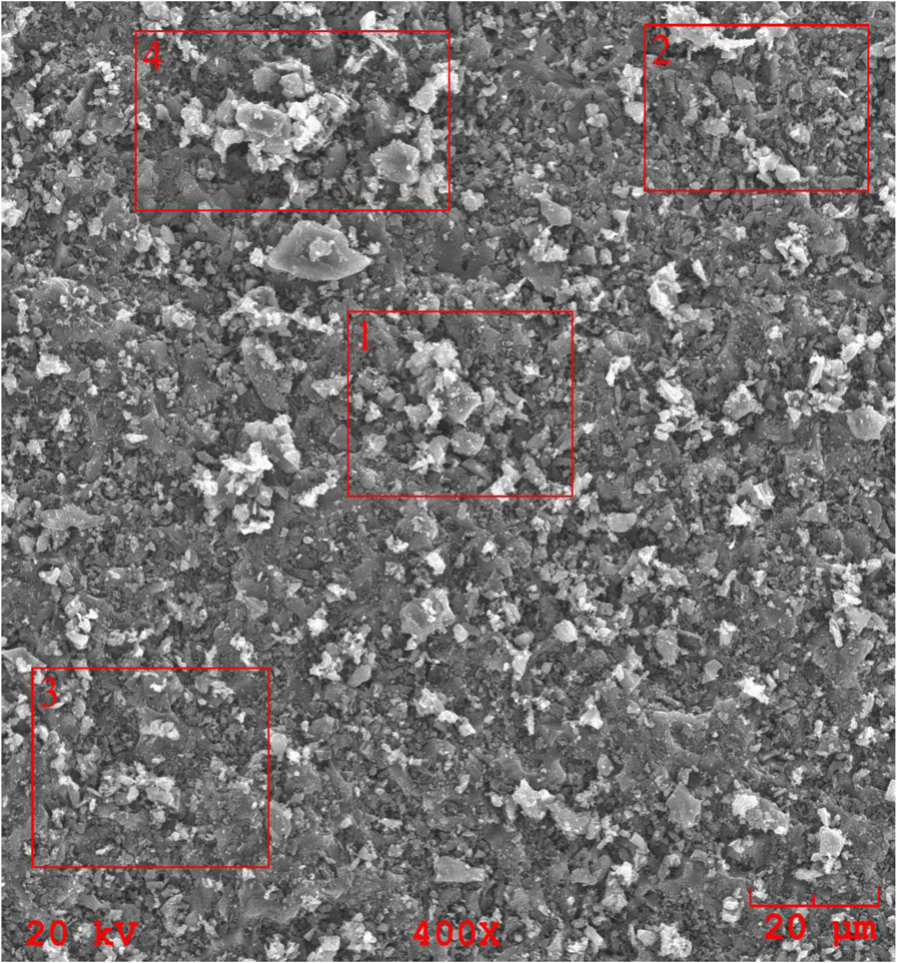
Micrographs of AC2 taken by SEM. Highlighted squares indicate points for EDS

**Fig. 3:**
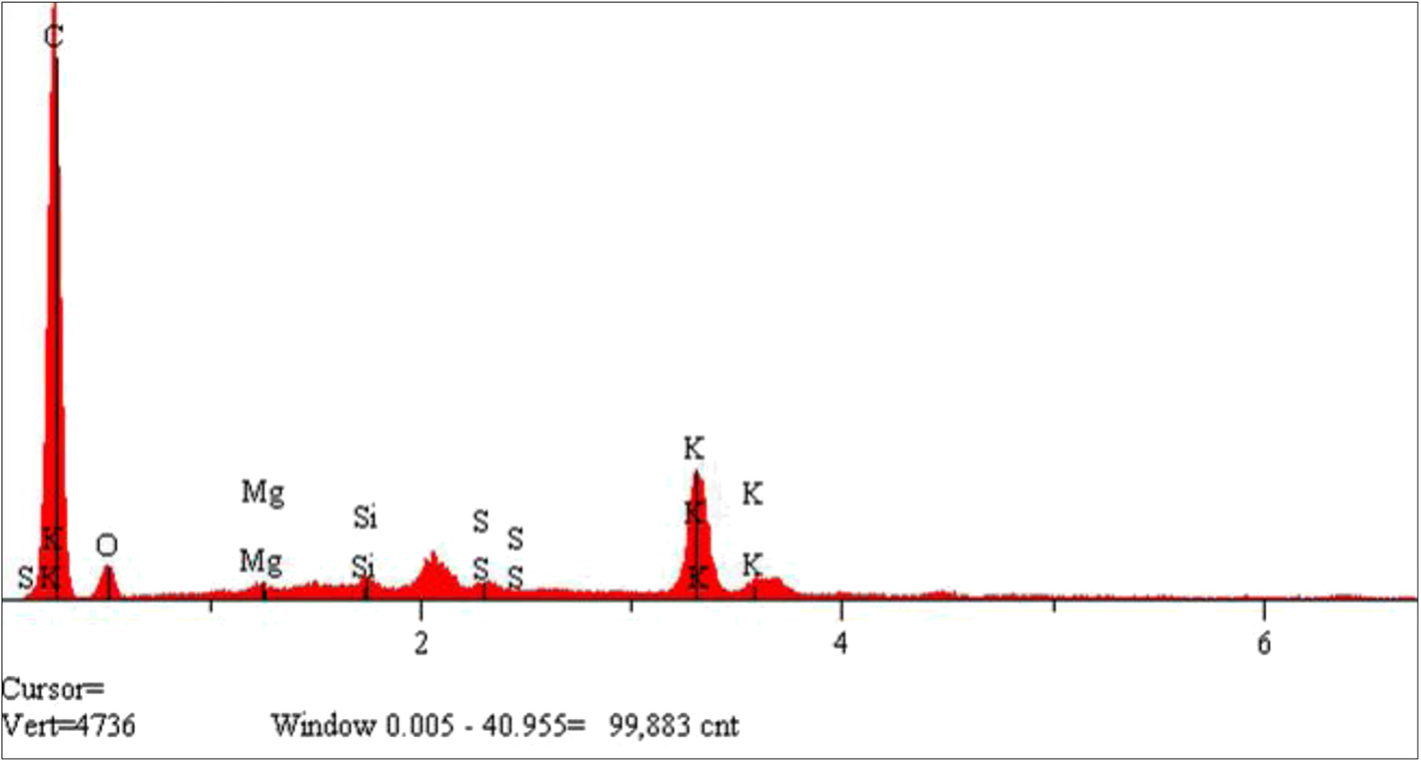
AC2 spectrum obtained by EDS

Figure 2 shows a scanning electron microscopy (SEM) image of the sample. The image of absorbent was enlarged 400 times, showing a homogeneously porous structure. With EDS (energy dispersive X-ray spectroscopy) coupled with SEM, a semiquantitative analysis of the chemical elements present in the structure of the obtained charcoal was possible, through the four points highlighted in the picture. EDS (Fig. 3) shows the most intense bands corresponding to elements C, O and K that can be considered as the natural species present in the bark of the Brazil-nut and that may influence the processes of ion exchange when charcoal is used as adsorbent. Other elements were also found in lower concentrations such as Mg, S and Si, as seen in Table 3. The amount of silicon found in this absorbent has a significant influence on the values of ash and moisture.

**Table 3:** Chemical composition of AC2

Elements	Point 1	Point 2	Point 3	Point 4
C	66.446	69.008	69.872	70.754
O	17.614	16.86	18.163	16.515
Mg	0.352	0.414	0.327	0.269
Si	0.439	0.401	0.541	0.493
S	0.628	0.65	0.511	0.503
K	14.521	12.668	10.586	11.465
Total	100	100	100	100

### X-ray diffraction

**Fig. 4:**
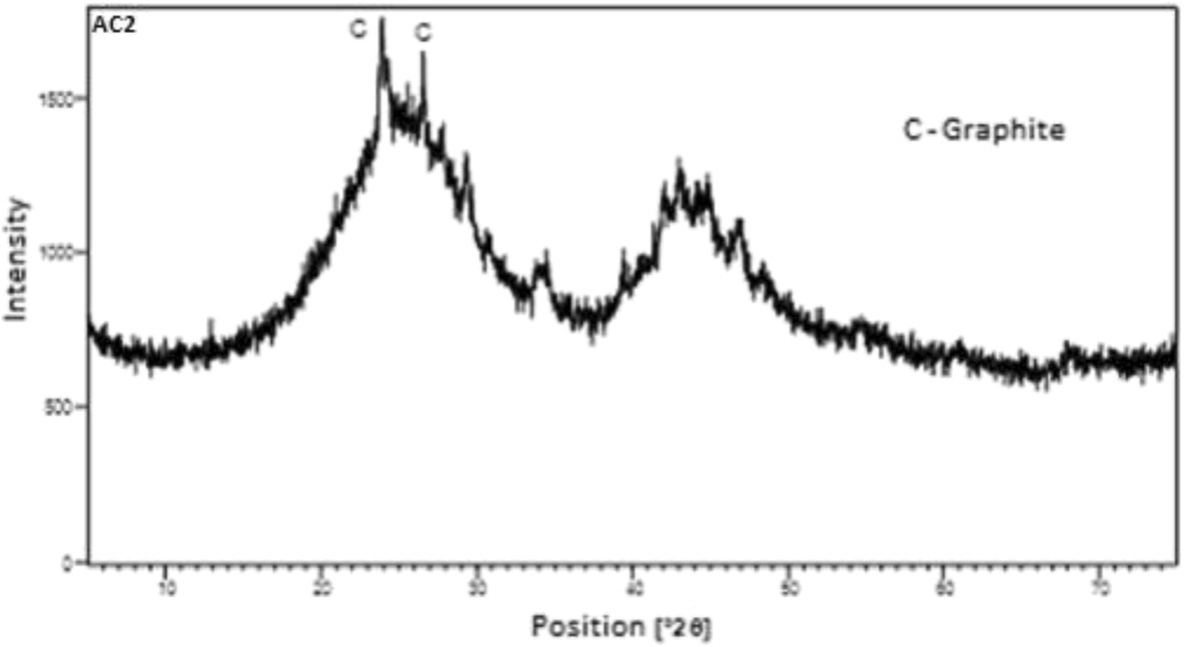
X-Ray diffraction of AC2

In the X-ray diffraction pattern shown in Fig. 4, it is observed that the dominant phase is mineral graphite (carbon graphite) with characteristic peaks in the range of 23.89 to 26,58 °. The amorphous materials (non-crystalline) are presented in large quantities, which can be observed in the elevation of the bottom line.

### Surface functional groups

**Fig. 5:**
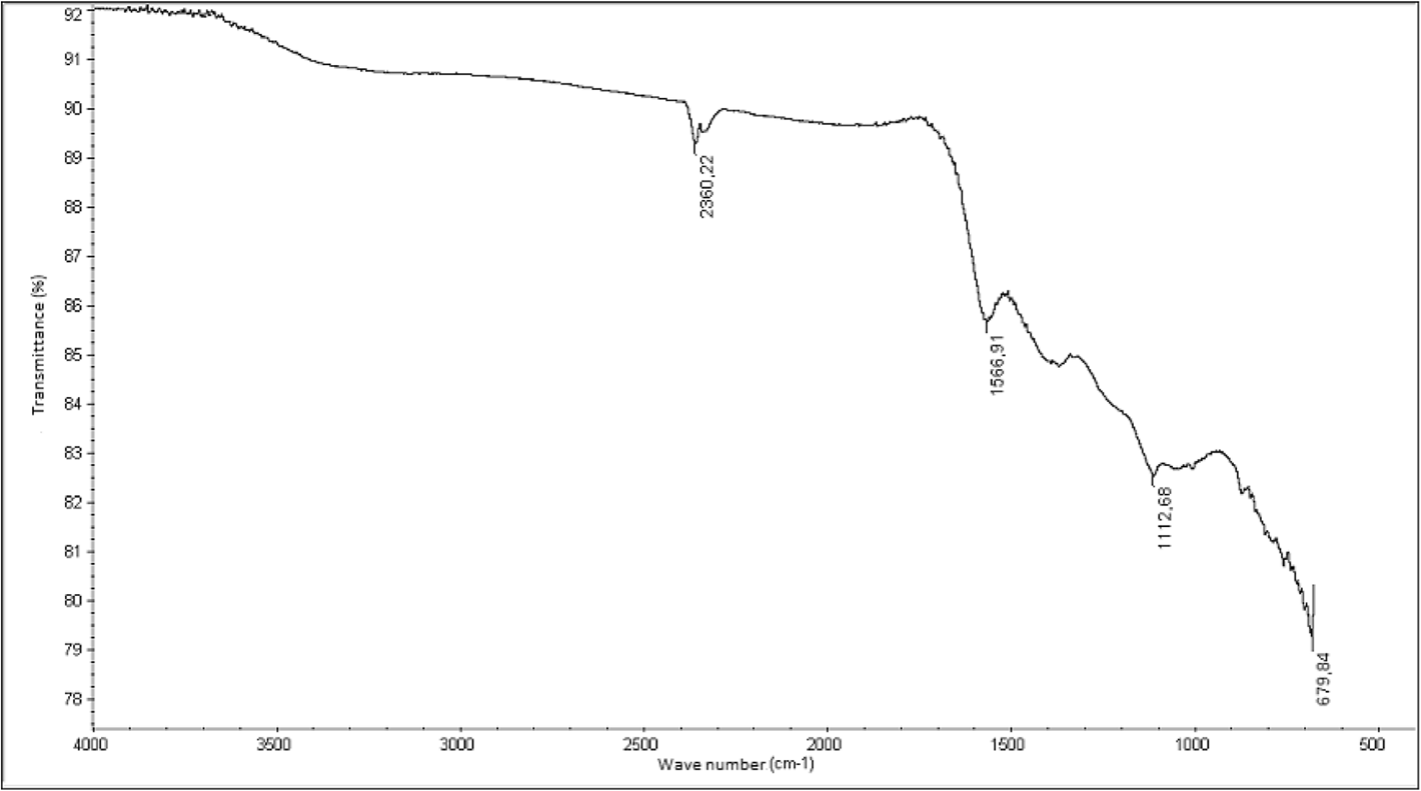
FTIR spectrum of AC2

To determine the functional groups methods such as Fourier transform infrared spectroscopy (FTIR) and Boehm titration were adopted. Table 4 shows the results from the Boehm titration of AC2. Using Equation 1, it was possible to quantify the percentage masses of acidic surface functional groups in charcoal, where it was possible to identify larger amount of phenolic hydroxyl groups (78.48 %) compared with carboxylic groups (17.91 %) and lactones (3.48 %). According to Liu et al. (2007) [26], phenolic hydroxyl groups and lactones dissociate at higher pH values than carboxylic groups. Observing FTIR spectrum (Fig. 5), the absorption bands around 1710 to 1760 cm^-1^ indicate the peaks relating to carboxyl groups (-COOH); the band from 1340 to 1480 cm^-1^ indicating the presence of phenolic hydroxy groups (-OH); around 1675 cm^-1^ to 1775 cm^-1^ indicating the presence of lactone groups (-OOR) and quinones (Bansal and Goyal, 2005) [8].

**Table 4:** Results of Boehm titration

Functional groups	MASFG^a^ (%)	Deviation
-COOH	17.91	0.023
-COOR	3.62	0.037
-OH	78.48	0.003

^a^MASFG: mass of acidic surface functional groups

### The influence of pH on Cu (II) adsorption

The values of adsorbed metal (Qe) and the removal efficiency (R%) of the metal in the study were obtained by equations (2) and (3) respectively. During the procedure it was observed that the removal percentage of the Cu (II) was proportional to the increase in the pH range, as seen in Table 5, wherein all assays showed the removal percentage above 90 %, with the exception of pH in the range 6.01, in which occurred copper hydroxide precipitation, Cu (OH)_2,_ which is not interesting for this process. In this case, pH equal to 5.09 is considered best suited to continue adsorption study of the Cu (II) with AC2 by being the natural range of synthetic solution and have higher metal removal percentage 96.42 %.

**Table 5:** The influence of pH on Cu (II) adsorption

pH	A C_e_(mg L^-1^)	B C_e_(mg L^-1^)	C_e_ (mg L^-1^)	MSE^a^	Q_e_ (mg g^-1^)	R (%)
3.04	2.33	2.11	2.22	0.11	3.75	95.56
4.01	2.06	1.79	1.92	0.13	4.44	96.15
5.09	1.85	1.73	1.79	0.06	4.53	96.42
6.01	2.18	3.05	2.61	0.43	4.15	94.77

^a^Mean standard error

### Influence of AC2 diameter of the particles in the Cu (II) adsorption

The influence of particle diameter is an important parameter, since the variety of adsorbent materials has a better adsorption capacity with a smaller particle size, due to the opening of tiny channels which, consequently, directly influence the contact surface between the adsorbent and the adsorbate in solution [27]. The effect of particle diameter on the AC2 in Cu (II) adsorption is presented in Table 6. It is noticed that the removal efficiency for the three granulometric ranges chosen for this process has been considered, since all the results were above 90 % of removal of Cu (II). Therefore, the intermediate range of 0.595 < D < 1.19 mm was chosen for subsequent adsorption experiments.

**Table 6:** Influence of AC2 diameter of the particles in the Cu (II) adsorption

DIAMETER	A C_e_ (mg L^-1^)	B C_e_(mg L^-1^)	C_e_ (mg L^-1^)	MSE^a^	Q_e_ (mg g^-1^)	R (%)
D > 1.19 mm	1.39	1.83	1.61	0.22	4.09	96.77
0.595 < D < 1.19	1.64	1.48	1.56	0.08	3.59	96.87
D < 0.595	3.27	2.90	3.08	0.18	3.93	93.83

^a^ Mean standard error

### Influence of the contact time of AC2 in solution Cu (II) adsorption

The results are shown in Table 7, and it is observed that the influence of the variation in contact time (adsorbent/adsorbate) in the results of Cu (II) removal efficiency is expressive, because it is observed that very fast equilibrium adsorption is achieved, whereas for all the agitation times, the removal percentage of above 90 % was reached.

**Table 7:** Influence of the contact time of AC2 in solution in Cu (II) adsorption

T (min)	A C_e_(mg L^-1^)	B C_e_(mg L^-1^)	C_e_ (mg L^-1^)	MSE^a^	Q_e_ (mg g^-1^)	R (%)
1	0.74	1.07	0.91	0.16	4.696	98.19
2	0.56	0.52	0.54	0.02	4.637	98.91
5	0.77	0.70	0.74	0.04	4.746	98.53
8	0.99	1.09	1.04	0.05	4.536	97.92
10	1.36	1.07	1.22	0.14	4.121	97.57
20	1.52	1.22	1.37	0.15	4.365	97.26
30	1.24	1.40	1.32	0.08	4.362	97.36
60	1.06	1.17	1.12	0.05	4.589	97.77
90	1.73	1.58	1.66	0.08	4.449	96.69
120	2.12	1.97	2.04	0.08	4.643	95.92

^a^ Mean standard error

### Influence of the equilibrium concentration

The removal efficiency of the Cu (II) by AC2 according to the varying of the initial concentration are shown in Table 8, and the results showed values above 90 % for concentrations 20, 30, 50, 100, 150 and 200 mg L^-1^, indicating positively the choice of the concentration equal to 50 mg L^-1^ of Cu (II) for experiments previously described. It was observed that the reduction in removal efficiency for the concentrations 5 and 10 mg L^-1^ is due to the lower mass transfer flow in the early stages of adsorption mechanism, as suggested by Melo (2012) [27].

**Table 8:** Influence of the initial concentration of Cu (II)

C_i_ (mg L^-1^)	A C_e_(mg L^-1^)	B C_e_(mg L^-1^)	C_e_ (mg L^-1^)	MSE^a^	Q_e_ (mg g^-1^)	(R%)
5	0.85	0.65	0.75	0.09	0.419	84.99
10	3.48	3.29	3.38	0.09	0.651	66.16
20	0.45	0.69	0.57	0.12	1.877	97.16
30	1.43	2.84	2.14	0.70	2.732	92.87
50	3.34	3.02	3.18	0.16	4.386	93.64
100	0.36	0.35	0.36	0.01	9.274	99.64
150	0.47	0.79	0.64	0.16	13.999	99.58
200	2.79	4.43	3.62	0.82	16.774	98.19

^a^Mean standard error

## Conclusion

In this study, it was possible to obtain thermally activated carbon from the bark of the Brazil-nut, an absorbent with mesoporous characteristics and satisfactory values of specific surface area. Moreover, it was possible to identify the presence of phenolic, carboxylic and lactonic groups on the surface of charcoal by means of infrared spectroscopy (FTIR) and Boehm titration. The properties of AC2 were similar to those coals from other vegetable precursors. Thus, the bark of the Brazil-nut, an abundant bio-residue, becomes a good alternative for the production of low cost activated carbon to be used in adsorption processes.
